# Towards an understanding of oleate hydratases and their application in industrial processes

**DOI:** 10.1186/s12934-022-01777-6

**Published:** 2022-04-09

**Authors:** Sophia Prem, Carl P. O. Helmer, Nicole Dimos, Stephanie Himpich, Thomas Brück, Daniel Garbe, Bernhard Loll

**Affiliations:** 1 Werner Siemens-Chair of Synthetic Biotechnology, Dept. of Chemistry, Technical University of Munich (TUM), Lichtenbergstr. 4, 85748 Garching, Germany; 2 Institute for Chemistry and Biochemistry, Laboratory of Structural Biochemistry, Freie Universität Berlin, Takustr. 6, 14195 Berlin, Germany

**Keywords:** Oleate hydratase, Biocatalysis, Industrial biotechnology, Whole cell and enzymatic oleic acid transformation, Green chemistry, Protein engineering, Structure–function relation, Bioeconomy

## Abstract

Fatty acid hydratases are unique to microorganisms. Their native function is the oxidation of unsaturated C–C bonds to enable detoxification of environmental toxins. Within this enzyme family, the oleate hydratases (Ohys), which catalyze the hydroxylation of oleic acid to 10-(*R*)-hydroxy stearic acid (10-HSA) have recently gained particular industrial interest. 10-HSA is considered to be a replacement for 12-(*R*)-hydroxy stearic acid (12-HSA), which has a broad application in the chemical and pharmaceutical industry. As 12-HSA is obtained through an energy consuming synthesis process, the biotechnological route for sustainable 10-HSA production is of significant industrial interest. All Ohys identified to date have a non-redox active FAD bound in their active site. Ohys can be divided in several subfamilies, that differ in their oligomerization state and the decoration with amino acids in their active sites. The latter observation indicates a different reaction mechanism across those subfamilies. Despite intensive biotechnological, biochemical and structural investigations, surprising little is known about substrate binding and the reaction mechanism of this enzyme family. This review, summarizes our current understanding of Ohys with a focus on sustainable biotransformation.

**Supplementary Information:**

The online version contains supplementary material available at 10.1186/s12934-022-01777-6.

## Introduction

Adaption to the outer environment is a crucial factor for survival of living organisms. Many microorganisms have found a way to survive toxins by producing detoxifying small molecules or proteins. One example is the detoxification of free long chain fatty acids by microorganisms, which in free form could potentially destroy outer membranes causing lysis of protoplasts, subsequent leakage of proteins, cell-associated fatty acids as well as nucleic acids. This is prevented by the expression of enzymes called fatty acid hydratases [1–5], which are unique to microorganisms [6]. Moreover, long chain fatty acids can cause prevention of protein and amino acid uptake, particularly in gram-positive bacteria due to the inherent character of their cell membranes [2, 7–9]. Consequently, several microorganisms, which live in close contact to free fatty acids, are reported to express fatty acid hydratases as an adaption and defence to their outer environment [10].

Two functions of oleate hydratases (Ohys) for microorganisms are currently discussed. Crude oils such as oils from plants but also from the skin typically contain a certain percentage of free, unsaturated fatty acids [11, 12], which are toxic for microorganisms, and thus it is thought that they are being detoxified via Ohys. *Staphylococcus aureus* has been found to express a functional Ohy even though it does not synthesize unsaturated fatty acids. However, one of *S. aureus*’ natural habitats is the human skin, where the high abundance of free, unsaturated fatty acids leads to an evolutionary pressure.

An Ohy has been discovered in *S. aureus* (OhySa) that conveys resistance against palmitoleic acid. The hydroxylated form does not further convey toxicity and is not incorporated into the phospholipid membrane but is rather exported into the outer environment [13]. Recently, it was shown that OhySa are able to convert host cis*-*9 unsaturated fatty acids to their 10-hydroxy derivatives in human serum and at the infection site in a mouse neutropenic thigh model, suggesting that OhySa could play a role in immune modulation in *S. aureus* pathogenesis [14]. Furthermore, fatty acid hydratases have been reported to be involved in stress responses of microorganisms. In *Bifidobacterium breve*, the expression of a fatty acid hydratase increases stability against heat and solvents [15, 16].

Ohys only convert free, unsaturated fatty acids, which is rather unique. Usually, bacteria can take up exogenous unsaturated fatty acids, but not all are incorporated into their phospholipid layer [17]. Furthermore, it is not fully understood, where exactly Ohys act. They could either function in the cytoplasm or in the outer environment. For *S. aureus*, it has been reported that Ohys were found in vesicles, which were secreted from the cell in the presence of linoleic acid [18]. Furthermore, an Ohy from *Lactobacillus plantarum* was found to be a protein, bound to a membrane by electrostatic attachment and additionally it was reported that the conversion of linoleate to 10-hydroxy-*cis*-12-octadecenoic acid occurs at the periphery of the cell [19]. Since a few microorganisms are known to contain several oleate hydratases, a complementary effect of defence might apply [20–22]. Membrane-hydratases and secreted ones could serve as a first level of defence and additionally, cytoplasmatic fatty acid hydratases could complement the response mechanism.

**Scheme 1:**
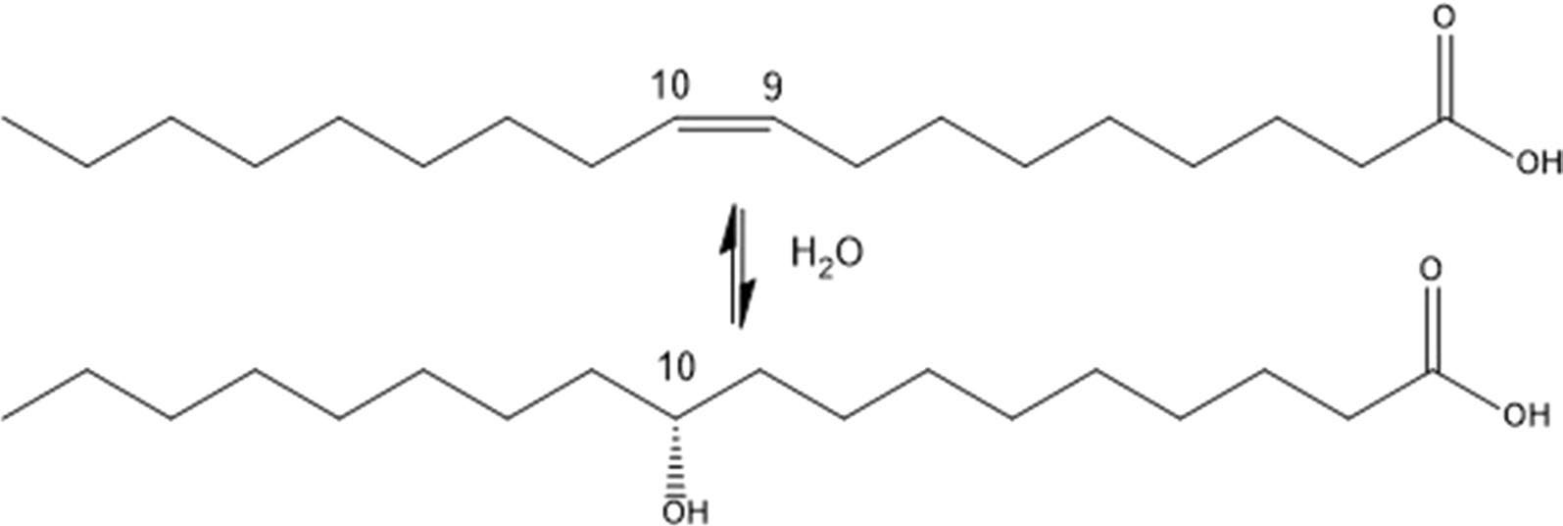
Hydroxylation of oleic acid to 10-(*R*)-hydroxy stearic acid as performed by Ohys

Fatty acid hydratases are able to hydroxylate unsaturated fatty acids. A plethora of fatty acid hydratases, which convert substrates with different acyl-chain length, ranging from C11:1 to C22:6, have been reported [20, 23–26]. Many fatty acid hydratases have low specificity, in respect to acyl-chain length, but demonstrate high regio- and stereospecifity. For instance, Ohys are regiospecific for the cis-9 C–C double bond position and enantiospecific for the 10-(*R)* isomer (Scheme 1).

Hydroxylated fatty acids have first been found in human steatorrhoeic faeces and since a standard diet does not contain such unusual fatty acids, it was assumed that microorganisms synthesize them in the gut [27]. This has subsequently been demonstrated, as a *Pseudomonas* sp. strain 3266 has been found to convert oleic acid to 10-(*R*)-hydroxy stearic acid (10-HSA; Scheme 1) [28]. Numerous other microorganisms, mostly discovered by investigating human or animal faeces, have been shown to produce 10-HSA [29–31]. Notably, 47 years passed by between the discovery of 10-HSA production of *Pseudomonas* sp. strain 3266*,* later found to be *Elizabethkingia meningoseptica*, and the purification and characterization of the responsible enzyme [24].

Prior to the discovery and characterization of the first Ohy, the first patent has been filed regarding the industrial use of an Ohy from *Streptococcus pyogenes,* including its direct homologues with more than 40% sequence overlap [32]. In an industrial context, oleate hydratases are of special interest, due to the high-value product 10-(*R*)-hydroxy stearic acid (10-HSA).

It was considered that 10-HSA can be a replacement for 12-(*R*)-hydroxy stearic acid (12-HSA), which is widely used in the chemical and pharmaceutical industry. As surfactant, 12-HSA is added to soaps and body washes. As molecule with emollient and thickening properties, it is used in skin creams and lotions. Other common applications are as an additive in grease, lubricating-oils and paints, in manufacturing PVC and as lubricants in synthetic or natural rubbers. Furthermore, it can be used as an adhesive and as a fine chemical in the food and pharmaceutical industry [33–35]. 10- and 12-HSA can additionally be converted into valuable secondary products using cascade reactions. Those include keto-fatty acids, estolides and wax esters [36, 37]. Advantages compared to similar products derived from petrochemicals are that 12-HSA can be manufactured from renewable recourses and it is considered as a low-risk compound [35]. In large industrial scale, 12-HSA is produced by chemical hydrogenation of castor oil mainly consisting of ricinoleic acid [38, 39]. For the hydrogenation of castor oil, either hydrogen and a metallic-catalyst such as Raney-Nickel is applied. Alternatively, a catalytic transfer hydrogenation without hydrogen can be performed. Often high pressure and temperatures are required to obtain sufficient yields of 12-HSA [40–43].

Recently, there is a call for more sustainability in the chemical industry, and the use of a biocatalyst could potentially support that demand. However, when producing 10-HSA using biocatalysts, free oleic acid is needed, which can be produced from oil of different types of renewable sources. This could either be plant-based oils such as high-oleic sunflower oil or when available in larger scales in immediate future, hydrolysed oil from microorganisms such as *Cutaneotrichosporon oleaginosus* [44] or free fatty acids produced from engineered bacteria [45]. As a result, there is less dependency on just one type of oil.

More and more new types of hydratases have been elucidated in recent years. This can be attributed to a growing interest in the industrial production of 10-HSA using biocatalysts. For industry and academia, an understanding of the precise mechanism of Ohys, including the role of a potential cofactor as well as substrate recognition is a fundamental prerequisite for protein engineering in respect to industrial application. Currently, the high requirements on the performance and process stability properties of these enzymes, which will be discussed in this review in detail, prevent their application in industrial processes. Successes in protein engineering are only achieved steadily and this can be attributed to many open questions regarding substrate and cofactor binding and the mechanism. Additionally, low substrate and product solubility hinder the appropriate capturing of enzymatic kinetic parameters. These struggles and ways to overcome them to establish well-functioning and stable Ohys will be the topic of this review. Moreover, we are going to discuss sequence specific differences within the Ohy families, potentially leading to differences in the catalytic mechanism.

## Architecture of Ohys

To date, there is very limited structural information available for Ohys (see Additional file 1: Table S1). Structural characterization of Ohys from only five different organisms was performed so far [26, 46–49]. For the sake of understanding, we will employ a uniform terminology for the description of the discussed Ohys. All Ohys will be referred to Ohy and the first two letters of the organism name of origin. We will mainly discuss: the Ohy of *Rhodococcus erythropolis* (OhyRe; Uniprot: T5I9M6), Ohy of *Staphylococcus aureus* (OhySa; Uniprot: A0A0D6GJV1), Ohy of *Lactobacillus acidophilus* (OhyLa; Uniprot: Q5FL96), Ohy of *Stenotrophomonas* sp. KCTC 12332 (OhySt; Uniprot: A0A126NKL7) as well as Ohy of *Elizabethkingia meningoseptica* (OhyEm; Uniprot: OLHYD).

Currently the structures cover three HFam families of the in total 11 Ohy families [25]. A superposition of the available crystal structures clearly reveals a higher similarity of structures within one clade of HFam families compared to lower similarity between families (see Additional file 1: Tables S2 and S3). Two structures of the HFam2 family, OhyLa as well as OhySa are available, which superimpose with a root mean square deviation (rmsd) of 1.1 Å (see Additional file 1: Table S3). In contrast, the superposition of the overall architecture of Ohys belonging to different HFam families, is significantly different with higher rmsd values (see Additional file 1: Table S3).

**Fig. 1:**
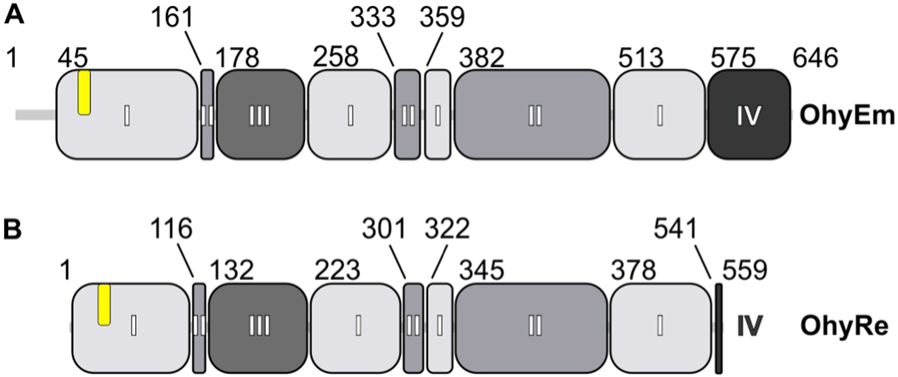
Schematic domain architecture of OhyEm and OhyRe. **A** Domain architecture of OhyEm coored in grey-shading for domain I to domain IV. In yellow marked the position of the Rossman signature motif. **B** Domain architecture of OhyRe with identical grey-shading for its domains as in OhyEm

**Fig. 2:**
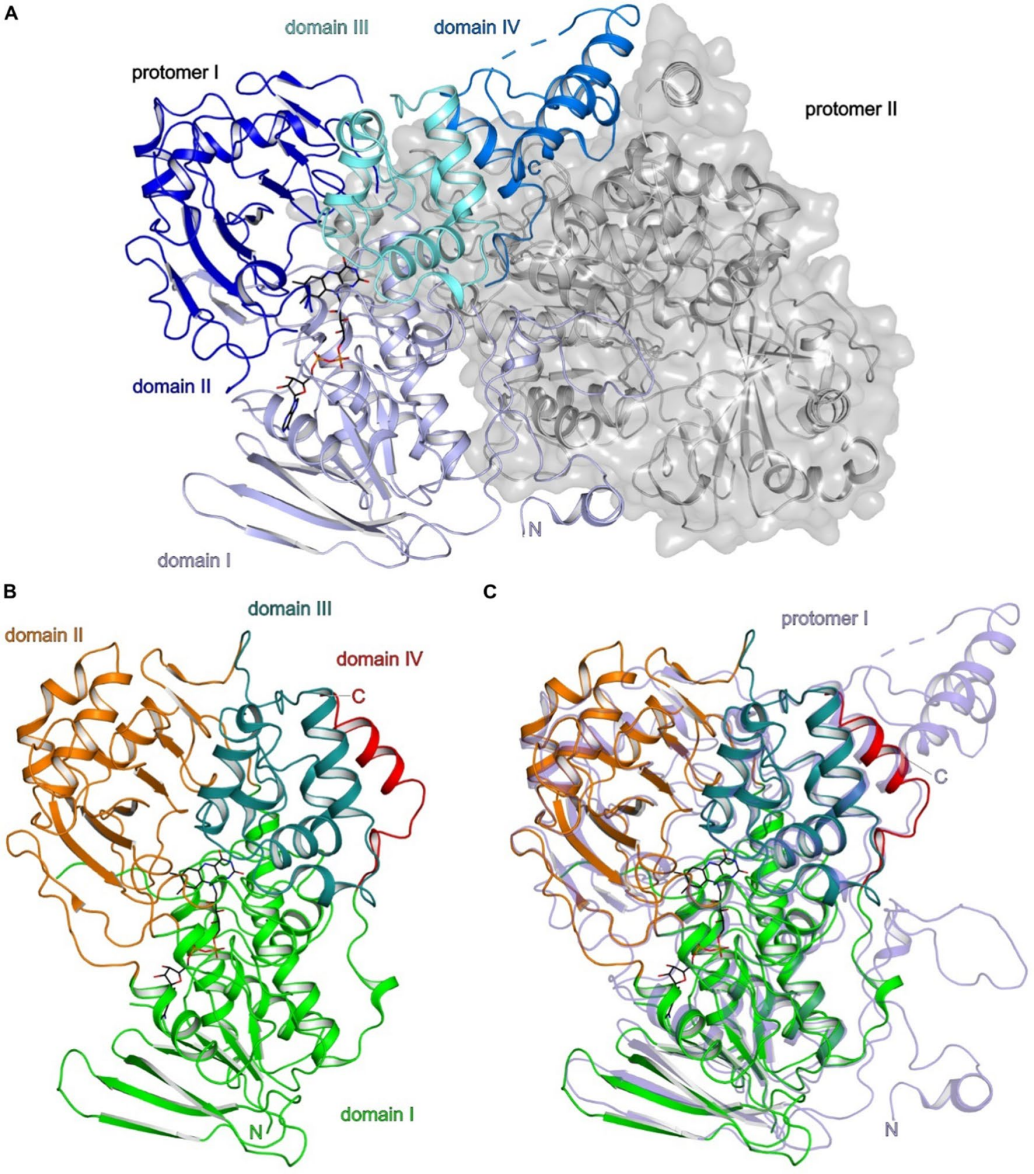
Overall structure of OhyEm and OhyRe. **A** Proteins are shown in cartoon representation. Dashed lines indicate un-modelled loop regions. Domain organization of OhyEm (PDB-ID: 4uir; [46]): Protomer I is shown in cartoon representation: Domain I in light blue, domain II in blue, domain III in deep teal and domain IV in marine. Protomer II is shown in a transparent surface representation and cartoon representation depicted in light gray. The FAD cofactor is shown as black stick representation. **B** Domain organization of OhyRe (PDB-ID: 5odo; [48]): Domain I in green, domain II in orange, domain III in deep teal and domain IV in red. The shown FAD cofactor is derived from the superposition with OhyEm. The FAD is depicted in black stick representation. **C** Superposition of protomer I of OhyEm and OhyRe in identical orientation as in panel B. One protomer of OhyEm is shown in light blue. The terminal extensions of OhyEm are clearly visible on the right site of the panel. Figures were prepared with PYMOL (Schrödinger Inc.)

The reported structures provide interesting insights into the binding of flavin adenine dinucleotide (FAD) and substrate binding sites as well as the different oligomeric states of Ohys. Common for all HFam families are three core domains (Fig. 1), but some subfamilies have additional N- and C-terminal extensions (Figs. 1, 2). Based on the available structural and biochemical information, Ohys can occur as monomers or dimers. OhyLa, OhyEm, OhyLa, OhySa and OhySt, all members of the HFam2 or HFam11 family arrange as dimers. In contrast, OhyRe is a monomeric enzyme belonging to HFam3. For dimeric Ohys common are N- and C-terminal amino acid sequence extensions, which are not present in the sequence of monomeric OhyRe [48] (Figs. 1, 2). These noticeable differences in the overall structure as well as in different oligomerization states of Ohys (Figs. 1, 2) could hint to variations in co-factor binding or substrate recognition. In the following, differences and similarities between various members of Ohy families with a focus on the domain arrangement, substrate and FAD-binding will be discussed.

### Overall structure

In general, all Ohy structures are composed of three core domains (Figs. 1, 2), that are related to other FAD-dependent enzymes. In the structures of the dimeric HFam2 and HFam11 family members, the proteins fold in an α-helix N-terminal of domain 1, which is involved in stabilization of the dimers. In Ohys of the HFam3 family the α-helix N-terminal of domain 1 is absent and the protein is monomeric. Domain I is a mixed α/β domain composed of a parallel five-stranded β-sheet packed between two α-helices on one side and a three-stranded antiparallel β-sheet on the other side (Fig. 2). Domain I resembles a variant of the Rossmann fold. Domain II consists of an antiparallel β-sheet (Fig. 2) flanked by three α helices defining the cofactor- and substrate-binding site in conjunction with domain I. Domain III is exclusively α-helical (Fig. 2) and its fold is structurally related to monoamine oxidases [50]. Together, domain II and III form a tunnel to guide the substrate into the active site. The C-terminal domain IV differs in size and if extended, contributes to the dimer interface (Fig. 2A, C). Domain IV undergoes a large conformational change upon substrate binding [51], suggesting a role of domain IV in substrate recognition in conjunction with domain II and III. Notably, the most significant structural differences are found for domain IV of all known Ohys, which could be caused by the size of domain IV and/or its involvement in substrate recognition. Hence, cofactor recognition and binding play a crucial role for Ohy activity in the different families implementing different catalytic pathways. Therefore, the role of cofactor binding will be discussed in the next paragraph.

### Functional role of FAD in Ohys

Ohys are lyases, which don’t necessarily require a redox-active cofactor. However, all known Ohys display a strictly conserved Rossmann-fold or Rossmann-fold like secondary structure motif [51], which are specific for binding of FAD or nicotinamide adenine dinucleotide phosphate NAD(P)H. In flavoproteins, FAD can either be bound covalently or non-covalently [52]. In the case of non-covalent binding, van-der-Waals and ionic interactions play a crucial role. As a result, FAD is bound via an on–off mechanism, that depending on the strength of binding can be more or less profound. Upon dilution, flavin molecules can be released from a protein even when they have picomolar binding-affinity [53]. In Ohys, the FAD is non-covalently bound to the protein. Therefore, binding of FAD induces a conformational change in domain I, which leads to closure of the FAD-binding site and enfolding of the FAD [46, 47].

All known Ohys strictly require FAD for functioning even though the FAD is likely not to function as a conventional redox cofactor known from other enzyme families [54]. The function of the FAD cofactor in Ohys is still under debate and could likely play a role in the polarization of the substrate, involvement in substrate binding or the stabilization of reaction intermediates [55, 56]. A merely structural role of FAD cannot be completely excluded and might contribute to stabilization of the protein. Thus, the crucial cofactor binding for structural integrity and function of Ohys remains an elusive question until now and hampers industrial approaches so far.

For most industrial processes, heterogeneous catalysis is the most common and preferred method. For economic reasons, enzymes are often preferred to be immobilized on solid supports [57–59]. However, each cycle of reuse induces a new equilibrium between medium and enzyme and thus over time, part of the cofactors can be lost, particularly in those enzymes with low binding affinity. This applies to Ohys, since they have weak binding affinity towards FAD [26, 48]. This leads to either partial or complete loss of FAD and activity. OhyRe loses both cofactor and activity, and OhyEm has only 86% of cofactor load [46]. OhyLa has been reported to lose FAD after extensive washing on an ion-exchange or affinity column and after gel-filtration [26]. In former immobilization experiments with OhyEm, a loss of activity after each round of reaction has been observed. The loss of FAD might be a possible explanation [37]. Thus, elucidating crucial amino acids for binding of FAD would aid in engineering the enzymes towards optimized variants, with a higher affinity towards FAD.

**Fig. 3:**
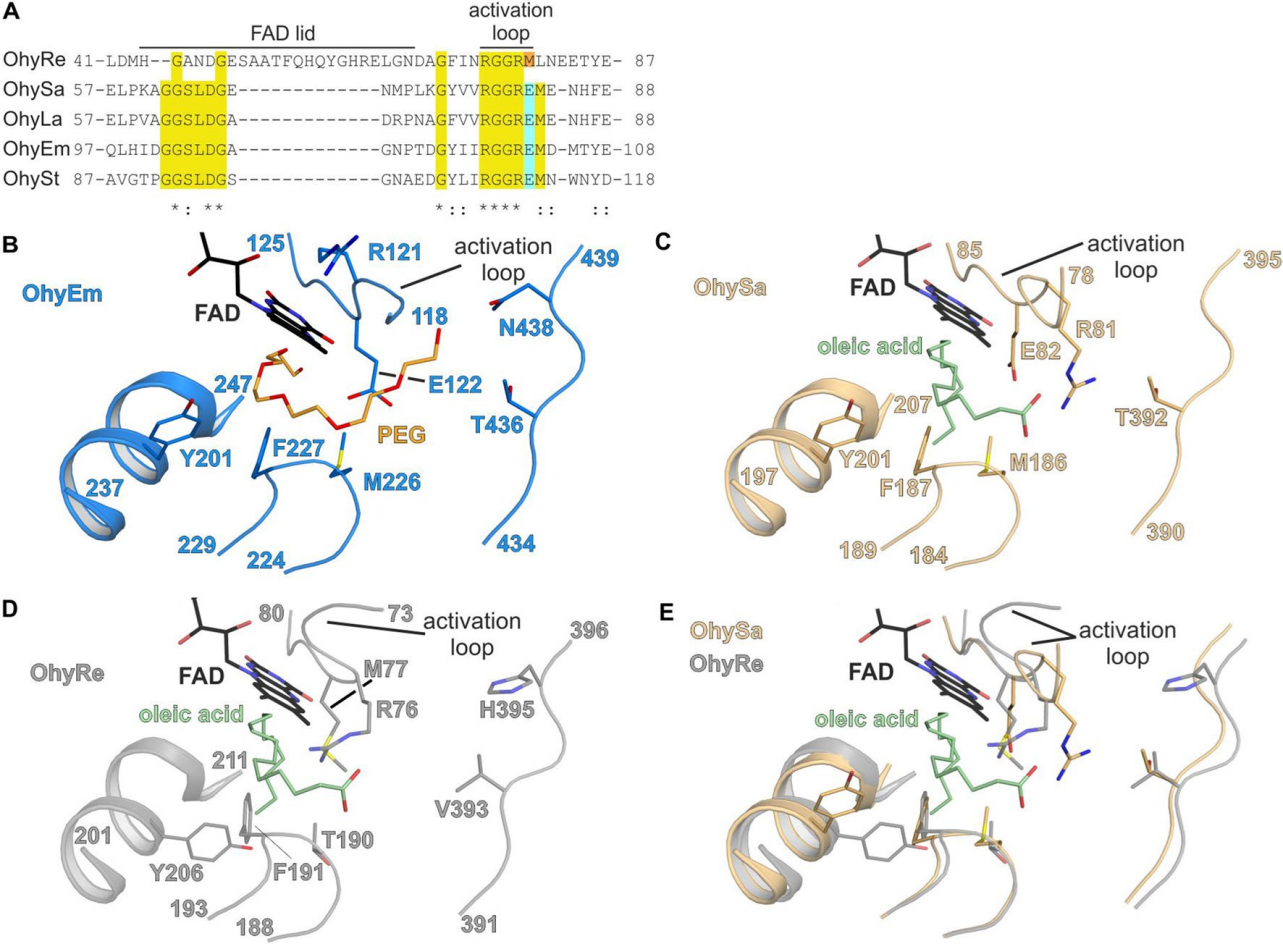
Sequence conservation of the FAD lid and the activation loop and architecture of the active site of OhySa, OhyEm, and OhyRe. **A** Amino acid sequence alignment of OhyRe (Uniprot: T5I9M6), OhySa (Uniprot: A0A0D6GJV1), OhyLa (Uniprot: Q5FL96), OhyEm (Uniprot: OLHYD), OhySt (Uniprot: A0A126NKL7) restricted to the FAD-lid and the activation loop. Conserved residues in lid and activation loop are highlighted by yellow background. The catalytic residue in the loop is highlighted with a light blue or light orange box, respectively. Highly conserved residues are indicated with asterisk, moderate conservation with two points, low conservation with one point. Primary sequences of Ohys were aligned using Clustal Omega [60]. **B** Active site of OhyEm (PDB-ID 4uir; [46]), shown with catalytic important residues. The bound PEG molecule in close proximity of the active site is shown in orange. Structural elements shown in cartoon representation. **C** Active site of OhySa (PDB-ID: 7kaz; [47]) shown with important residues lining the active site. The ternary complex of OhySa with bound FAD and oleate was obtained with the OhySa variant E82A. For clarity, we have computationally re-introduced the wild-type situation. FAD, oleic acid and indicated residues shown in stick representation. **D** Active site of OhyRe (PDB-ID: 5odo; [48]) shown with important residues lining the active site in stick representation. The shown FAD cofactor and oleic acid were obtained by a superposition of the OhySa structure A and derived from the superposition with the structure of OhySa. **E** Superposition of the active site of OhyRe and OhySa

In domain I, the FAD-binding pocket is defined by the Rossman-fold as well as a lid region, that undergoes a conformational change upon binding of FAD. Latter conformational change ultimately leads to a closure of the FAD-binding pocket with the lid segment in close proximity to the isoalloxazine ring, the diphosphate function as well as the ribose of FAD. Interestingly, the length of the lid segment differs between HFam family members. The lid segment has a length of 17 amino acids in all Ohys so far structurally characterized, with just one exception for OhyRe (Fig. 3A). Here, the lid is significantly longer with 27 amino acids (Fig. 3A). One might ask, whether this could be a structural feature of members of the HFam3 family. Hence, we aligned all available amino acid sequences of subfamily HFam3, available in the assembled “hydratase engineering database” [25]. Our sequence analysis clearly revealed that all HFam3 family members contain an extended lid with a length of 27 or 25 amino acid residues, indicating a distinct structural feature of this family. Despite its length, in all structures, the lid segment contains the highly conserved signature motif GGXXXG (X any amino acid; Fig. 3A). Notably, in HFam3, the motif is altered to GXXXG. Concomitant with FAD-binding, a loop region, termed “activation loop” by Radka et al*.* [47], undergoes a large conformational change (Fig. 3E). As consequence of FAD-binding, the activation loop almost rotates by 180°, otherwise it would lead to a steric clash. In the FAD-bound state, the activation loop is in proximity to the isoalloxazine function of FAD and secondly, it pre-shapes the substrate binding pocket for the approaching substrate. In the structure of OhySa, the activation loop comprises residues from 78 to 83 (Fig. 3A,). Notably, the catalytic E122 of OhyEm as well as E82 of OhySa are located within latter activation segment (Fig. 3B, C).

**Fig. 4:**
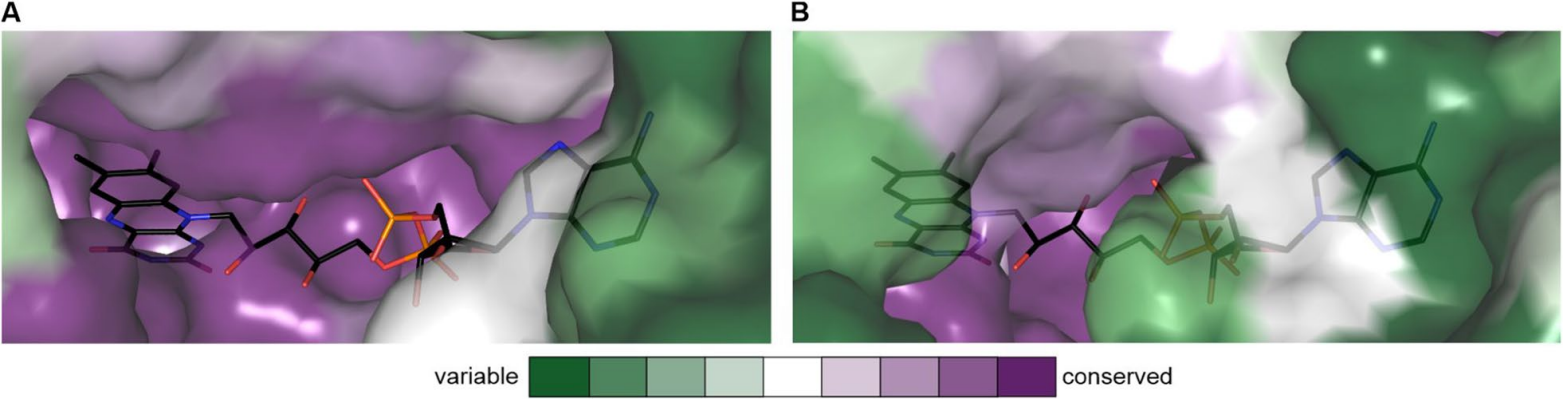
Conservation of FAD-binding pocket. **A** Surface representation of OhyRe (PDB-ID: 5odo; [48]) with conservation of residues from variable to conserved as indicated in the legend. The shown FAD cofactor is depicted as black stick representation and derived from a superposition with OhySa. In the structure of OhyRe, the FAD-lid is disordered and could not be modelled. **B** Surface representation of OhySa (PDB-ID: 7kaz; [47]) with conservation of residues from variable to conserved as indicated in the legend. The FAD-lid covers the bound FAD molecule. Conservation of Ohys was calculated with the Consurf server [61]

Analysing the sequence conservation of the FAD-binding pocket, clearly reveals a very high degree of sequence conservation of the surface shaping the pocket (Fig. 4). The observed differences in affinity towards FAD could be likely attributed to differences in the length and amino acid sequence pattern of the FAD lid, which have consequences for the conformational flexibility of the lid region. Such conformational flexibility is also structurally reflected. For instance, in the structure of OhySa, a weaker electron density compared to the protein was interpreted as a not fully occupied FAD and fragmented electron density was observed for the lid region, supporting an inherent flexibility of the lid region [47].

### Substrate binding

Recently, the crystal structure of an OhySa variant bound to oleate and FAD was reported [47], giving insights into the active site configuration. Previously, structures of Ohys from other organisms were reported with polyethylene glycol (PEG) molecules, originating from the crystallization experiment, bound in the cavity in proximity to FAD. It was proposed, that the PEG might resemble the substrate [46, 47]. The structure of OhyLa was reported with a bound linoleic acid in domain IV, distinct from the active site [26].

**Fig. 5:**
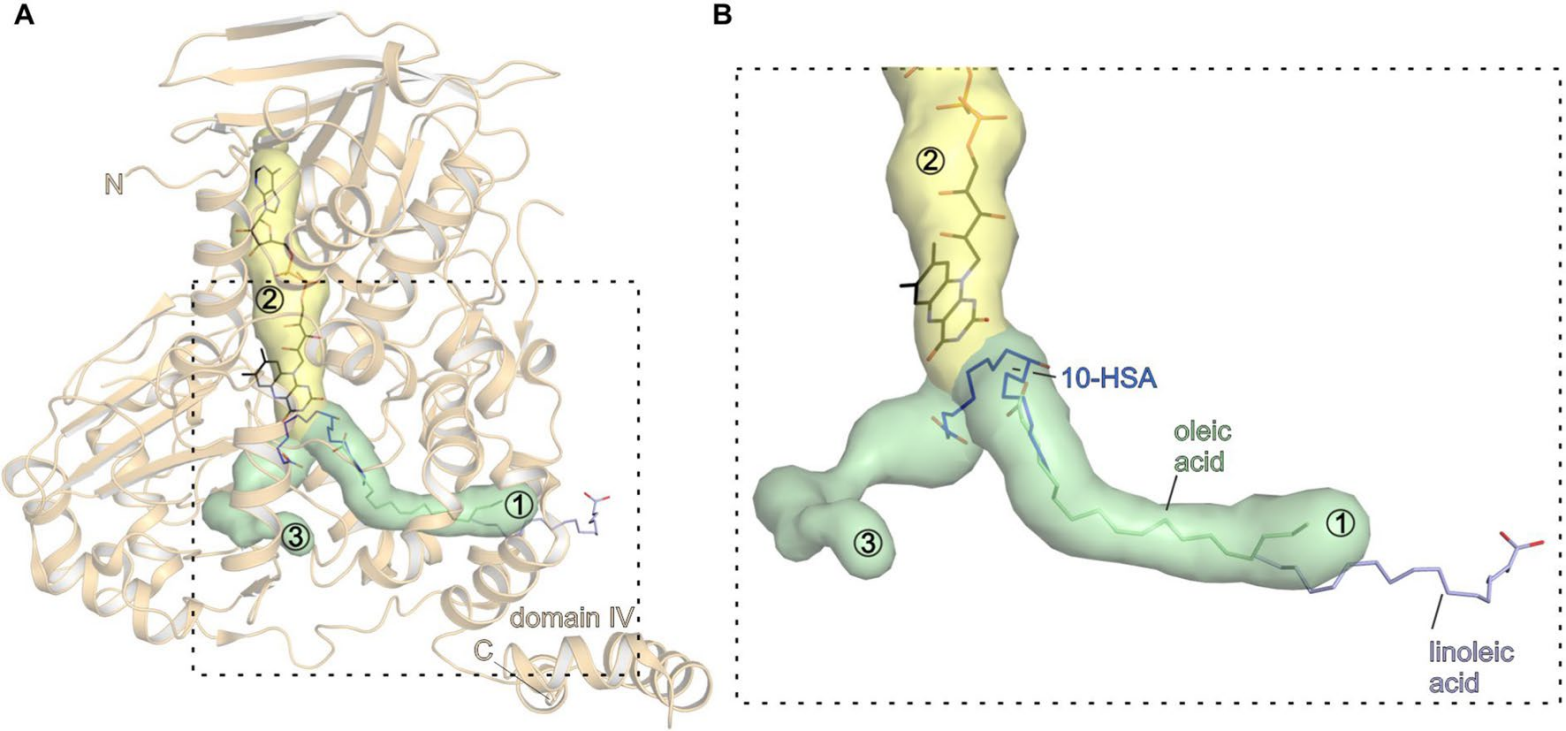
Substrate, product tunnel and FAD-binding site in OhySa. **A** OhySa in light orange cartoon representation and the predicted substrate, product and FAD cavities in surface representation in yellow and green. The substrate/product channel from the exterior of the protein towards the FAD is shown in green and numbered “3”. The cavity with bound FAD is depicted in yellow and numbered with “2”. A side channel in vicinity of the FAD cavity is labelled with “3”. The tunnels were calculated with Caver 3.0 [62]. FAD is shown in black stick representation. Oleic acid (PDB ID: 7kaz; [47]) is shown in light green stick representation; 10-HSA bound to OhySa (PDB ID: 7kaz; [47]) is shown in dark blue stick representation as well as linoleic acid bound to OhyLa (PDB ID: 4ia6; [26]) is shown in light violet stick representation; Dashed box displays magnification area as shown in **B**. **B** Magnification of dashed box in **A** with the protein omitted. The ligands linoleic acid, oleic acid and polyethylene glycol are depicted as in **A**. The ligands guide the substrate channel from the distal end of the channel to the proximal catalytic cleft close to the FAD

Superposition of all bound ligands in the structure of OhySa (Fig. 5) illustrates, that a substrate channel is built from the distal part (linoleic acid) to the active site (10-HSA). Oleic acid occupies this tunnel in between linoleic acid and 10-HSA (Fig. 5). Mainly domain III and domain IV build up the ligand channel, which is lined by hydrophobic amino acids, allowing the mainly hydrophobic substrate to diffuse into the active site niche. The role of the flexible domain IV in the catalytic cycle of Ohys remains elusive. Interestingly upon binding of ligands a conformational shift of the domain IV is observed in the structure of OhyLa [26]. Notably, for the monomeric OhyRe, belonging to the HFam3 family, the domain IV is significantly reduced in size compared to dimeric Ohys. Moreover, calculation of potential ligand channels in the structure of OhyRe in its apo state was not possible, indicating that the substrate channel in the structure of OhyRe is blocked or not yet formed. Analysis of the OhyRe structure reveals that α-helices of domain III are in closer proximity to each other, narrowing the channel. In addition, a number of amino acid side chains with hydrophobic character point into the putative channel. Interestingly, many of these residues are conserved or at least similar to OhySa. In the product or substrate bound state of the variant OhySa E82A, the α-helices of domain III and side chain rotamers adopt a different conformation, opening a channel in direction towards the FAD molecule.

Further, it remains unknown how the substrate persuades along the approximately 30 Å long ligand channel from the protein exterior towards the catalytic site. A hypothesis could be that the substrate diffuses through the channel towards the active site. Yet another possibility could be a partial opening of the protein with subsequent binding of the substrate. The initially bound substrate could then further diffuse along the channel. However, passive diffusion of the substrate seems to be rather atypical for such long ligand channels, since also the product needs to diffuse through this channel to the exterior of the enzyme and passive diffusion would also not be time efficient in the catalytic process. Moreover, a pure diffusion mechanism might be unlikely since the chemical structure of oleic acid with its C9 *cis* double bond makes the substrate rather rigid. In absence of substrate or product, water molecules should, at least partially fill the empty ligand channel in the apo state of Ohys. Latter water molecules need to be expelled upon substrate binding or substrate diffusion along the cavity. Moreover, we detected a side opening in the structure of OhySa (Fig. 5), through which water molecules could be pushed out by the moving substrate on its trajectory towards its binding site. This shorter side cavity is mainly lined by hydrophilic amino acids and was described in the structure of OhyEm [46].

Interestingly, linoleic acid binds to OhyLa with the carboxylate function facing outward and the hydrophobic poly-carbon tail threaded into the channel formed by domain IV ([26] Fig. 5B). It should be noted, that the electron density interpreted as linoleic acid is weak. A clear decision on the orientation of the carboxylate is very difficult and modelling of the carboxylate is based on the observation of Arg and Lys residues in the closer neighbourhood to the carboxylate. In contrast, in the structure of OhySa, the substrate oleic acid and the product 10-HSA are both bound with the carboxyl group facing inward (Fig. 5B). Additionally, the binding mode of the ligands is not thoroughly understood yet. Several amino acids lining the active site of Ohys need to rearrange during binding of the substrates. Especially R81 of OhySa is a crucial residue in binding of the substrate (Fig. 3B), since it acts as a block before the entry of the substrate and its site chain rotates about 180° upon FAD-binding. As a consequence, the guanidinium function of R81 points in the direction of the approaching substrate. Surprisingly, based on the structure of OhySa with bound oleic acid, the positively charged guanidinium function is not directly involved in recognition of the negatively charged carboxylate of oleic acid [47]. Given the chemical structure of oleic acid, only van der Waals interactions with the carbon skeleton are possible and only the charged carboxyl moiety could be sensed by the protein environment. One could imagine that for a stereospecific hydroxylation, the substrate would have to be held in a very precise, defined position to avoid any side reactions.

### Reaction mechanism

The overall architecture of the active site is well preserved within all structurally characterized Ohys. Nevertheless, substantial differences can be noted between the members of the different HFam subfamily members and will be discussed here. Based on the crystal structure and docking studies, a reaction mechanism for OhyEm was proposed, where Y241 initially protonates the double bond of the substrate. E122 activates a water molecule that can quench the carbocation [46, 56, 63]. Recently, a similar function was proposed for the catalytic E82 of OhySa (Fig. 3C) [47]. In contrast to the earlier proposed function, Y201 is hydrogen bonded to the backbone carbonyl of V505. Consequently, Y201 cannot donate a proton to the double bond. Conversely, our modelling data indicates that it is involved in a hydrogen bonding network including the hydroxylated oleate. A hydronium ion is stabilized by an α-helical dipole and a cation of an acidic proton of E122. Subsequently, the hydronium ion attacks the substrate. Upon release of FAD, the proton is released to the hydrated active site and in turn to the C10-hydroxylated product [47].

Instead of a glutamate in the active site at an equivalent position of E122 in OhyEm or E82 in OhySa, belonging to HFam11 and HFam2, respectively, a methionine residue is located in OhyRe, belonging to HFam3 family (Fig. 3E). We were puzzled whether a methionine residue within the activation loop could be a conserved feature within the HFam3. Amino acid sequence analysis of the “hydratase engineering database” [25] revealed a strict conservation of the methionine residue. Mutational studies of OhyRe variants M77E showed a drastically reduced hydroxylation activity compared to the wild type enzyme [48]. A plausible role of the methionine could be a stabilization of the emerging carbocation [64]. Given the fundamental difference in chemistry of glutamate *versus* methionine poses the question, whether Ohys belonging to the HFam3 family employ a different reaction mechanism. The family-specific patterns such as dimeric versus monomeric enzymes; differences in the length of the FAD-lid as well as different decoration of the active site niche could hint at different reaction mechanisms and explain the differences in substrate recognition. In summary, the observed differences might indicate a convergent evolution of Ohy families from different ancestors. Consequently, these observations path the way for a deeper understanding and implementation of Ohys in biotechnological pathways and will help to employ such enzymes for the chemical industry. In the following chapter a more detailed overview on biotechnological and industrial application of Ohys will be given.

## Industrial application—up and downstream innovation

Enzymes are currently used in a wide variety of industrial processes. These traditionally include the food, feed, polymer, leather and cosmetics sectors. Moreover, enzymes are also used as functional detergent additives and in organic synthesis of specialty chemicals [65]. However, not all enzymes make it into an industrial process for several reasons and overcoming those challenges is one of the major tasks of protein scientists. The performance and the costly development of processes are the main hurdles for using enzymes in an industrial environment [66].

### Measuring kinetic parameters

For many Ohys, enzymatically determined kinetic parameters have been published, and those could be used for industrial process simulations and cost evaluations. However, for Ohys they have to be considered with caution, as the substrate, oleic acid, is not fully miscible in water. Suspensions can be prepared by vigorous mixing, but oleic acid is not equally distributed, hence care has to be taken on using stock suspensions for enzymatically determined kinetic measurements. This is critical for the measurement of kinetic parameters but also for the reaction itself. When oleic acid is not brought into suspension, droplets are formed, lowering the access towards the catalyst and thus a decreasing yield, as observed by Jeon *et. al*., which could be resolved by more efficient mixing resulting in the formation of a suspension [67].

Furthermore, the pH and the temperature have effect on the formation of certain fatty acid species. At a pH lower than 6, fatty acids usually form crystals and at a pH between 7 and 9, they are in a 1:1 acid to soap ratio, which have extremely low solubility in water [68]. Ohys can have diverse pH-optima, OhyEm has two pH-optima at pH 6 and 8 [24], OhyRe at 7 [48] and Ohy from *Rhodococcus pyridinivorans* has an optimum at pH 5 [69]. It is unclear what kind of effects those fatty acid species have on the enzyme and the reaction. Additionally, 10-HSA is a product that has no solubility in water and depending on the experimental reaction environment, presence of certain surfactants or mixing speeds generates different forms of white aggregates in a solution. However, taking samples from a solution can only provide an initial overview due to misleading distributive effects and enzymatic kinetic measurements shall be performed in single reactions.

Also, 10-HSA has low solubility in many organic solvents. That is why full extraction of product and substrate can’t be achieved under certain extraction conditions and consequently their measurement is distorted. A common extraction solvent is ethyl acetate after acidification of the reaction solution, but also chloroform/methanol is used [48, 70]. However, the solubility of 10-HSA in those extraction solvents for analytical purposes has not been reported in most studies. Furthermore, several methods for purification of 10-HSA in a preparative scale have been performed. In one study, fractionation using acetone and acetonitrile has been performed at low temperatures resulting in a purity of over 99% [71].

Mostly, gas chromatography is the analysis method of choice and for that, derivatization of the fatty acids has to take place to reduce adsorption effects. This is achieved by methylation or silylation of the carboxy and hydroxy groups [72, 73]. Additionally, at present, non-derivatized 10-HSA cannot be commercially purchased and thus the standard has to be prepared in-house. For that, however, an internal standard is crucial, to evaluate the derivatization efficiency and evaporation effects. Only then, the instrument can properly be calibrated.

To sum up, for enzymatic kinetic measurements, which are important for industrial process simulations, several considerations are necessary. Only under certain conditions, appropriate enzymatic kinetic parameters can be determined. Those apply, when oleic acid is added purely to the reaction, when complete extraction of substrate and product is performed and the standards are prepared carefully. Furthermore, enzyme kinetics should be performed in single reactions and it is important to keep in mind that they are not comparable between enzymes and studies due to varying reaction conditions.

### Performance of whole-cell catalysts

The performance of a catalyst is crucial for every industrial process, in heterogenous catalysis the space–time yield (STY) can be around 1 to 10 kg L^−1^ h^−1^. However, when looking at biocatalysts, STYs can be decreased by up to 1000 times to around 0.001–0.3 kg L^−1^ h^−1^ compared to conventional processes [66]. While this certainly can be tolerated by the pharmaceutical industry with a need for enantiomeric purity and high-quality products, expensive processes for a final product, that is mostly used as an additive such as 12-HSA, will most likely not sustain.

An enzymatic industrial process can be installed in different modes. First, either wild-type or genetically engineered whole-cells can be used to convert oleic acid. The advantage here is, that no further purification of the enzyme is needed, only the extraction of fatty acids and the purification of 10-HSA. Usually, whole-cell conversions apply, when large gene clusters and cascades are involved in the formation of a product or if enzymes are not soluble or active when being isolated.

Recombinant *Escherichia coli* expressing an Ohy from *Stenotrophomonas maltophilia* has been used as whole-cell catalysts in a 1 mL scale leading to a STY of 12.3 g L^−1^ h^−1^, however, when they scaled-up to 1 L, the STY decreased to 8.2 g L^−1^ h^−1^, presumably due to the changed reaction conditions omitting the buffer and oxygen-depletion [67, 74]. Furthermore, the authors mention that genetically modified *E. coli* has a threefold higher formation rate than the wild-type strain. In other studies, the original organisms have been used, however the STYs were quite low compared to *E. coli*, except for one study with *Stenotrophomonas nitritireducens*, where 7.9 g L^−1^ h^−1^ was achieved [74]. However, the prolonged growth of *S. nitritireducens* and the maximum achievable cell concentration compared to *E. coli* were not considered. With *S. maltophilia* for instance, only 10 g/L of maximal cell concentration can be achieved compared to 100 g/L in *E. coli* in fed-batch cultures [75]. Thus, the authors concluded that *E. coli* as whole-cell catalyst is more advantageous compared to wild-type strains. Mass transport limitations are hurdles during reactions with whole-cell catalysts and this is particularly the case for enzymes converting bulky substrates such as Ohys [25]. One study aimed to overcome this effect by decreasing the route between catalyst and substrate. For that, the enzyme was targeted into the periplasm using a signal peptide. The whole-cell reaction using the periplasmatic enzyme resulted in a tenfold higher hydration rate compared to the cytoplasmatic reaction. It is known, that the redox-environment plays a role on the activity of Ohys but this has not been discussed [76].

Currently the highest reported STYs for producing 10-HSA with a whole-cell catalyst is 8–12 g L^−1^ h^−1^. For comparison, the production of acrylamide with nitrile hydratase, which is one of the most efficient whole-cell biocatalytic processes in the industry, gives STYs between 53 and 93 g L^−1^ h^−1^ [66, 77]. To reach such a level for the production of hydroxylated fatty acids, significant process optimization is required. However, using whole-cell catalysts also brings disadvantages particularly for this certain application. Free, unsaturated fatty acids might convey toxic effects on the whole-cell catalysts upon a certain concentration since the detoxifying fatty acid hydratases usually are expressed in the cytosol. Additionally, as already mentioned the mass transport of substrate and product is hindered by the membrane. Therefore, it is important to keep the fatty acid content under a critical toxic concentration and for the latter issue, organic solvents or surfactants such as Tween80 can be added. Those additives, however, can influence the energy metabolism within the cell, increase the costs of a process and might complicate the purification. So other strategies such as genetic engineering are investigated [78, 79].

Additionally, in the aforementioned studies, samples of the reaction medium were taken and extracted using organic solvents such as ethyl acetate. Consequently, endogenous fatty acids and hydrophobic molecules from the cells are extracted and appear as impurities in the final product. Whereas this might be no problem for industrial products, formulations for the pharmaceutical or cosmetic industry certainly have higher standards regarding the purity and more laborious downstream processing is required to further purify the product. Other possibilities are the filtration but this might come with a significant product loss since substrate and product might adsorb to the cell exteriors. In one study about whole-cell biocatalysis, 30% of loss was observed after downstream processing [67]. The high product losses due to using whole-cell biocatalysts can also be attributed to the faster saturation of extraction solvents due to hydrophobic molecules from the cell. Lastly, it is much harder to recycle whole-cell catalysts, particularly when the product is solid and centrifugal forces do not lead to a separation of product and catalyst.

### Performance of Ohys in lysates and pure formes

That is why in some cases, lysates or purified enzymes might be more desirable. Contrarily to some other fatty acid converting enzymes or other hydratases [80, 81], Ohys achieve high expression rates and solubility and don’t rely on stoichiometric amounts of FAD [56], which makes them excellent candidates for use in pure form or in lysates. Many screenings of activities of Ohy have been performed using lysates and lyophilized lysates [25], and a patent described the large-scale production of 10-HSA with lysate of *R. erythropolis* and *S. maltophilia* [82]. Furthermore, a pilot scale with cell-free extract has been performed using a variant of *Paracoccus aminophilus* with a STY of 22.5 g L^−1^ h^−1^ [83].

Since lysates are difficult to recycle and have weak stability; pure, immobilized enzymes are in some cases the method of choice. Additionally, immobilization can result in higher stability, increased activity and improved stereoselectivity and efficient recycling lowers the costs [84]. However, not all enzymes can be immobilized and recycled for several rounds in native form and not all products have good biocompatibility with the solid supports. Ways to overcome these challenges are on the way by developing novel supports and materials for immobilization and by using state-of the art technologies in the field of protein engineering [84].

One of the main issues for immobilization is the mentioned insolubility of 10-HSA in water and thus the catalyst cannot easily be separated by centrifugation. Furthermore, oleate is a hydrophobic molecule and thus attaches to certain materials used as solid supports. At present, only one study exists, where an Ohy has been immobilized. Several issues occurred while testing different kinds of support. The recovery of the product without harming the enzyme in form of cross-linked enzyme aggregates was not possible. To ease the separation of catalyst and product, magnetic beads were used, however that resulted in adsorption of substrate and product to the support. The magnetic beads were coated with a layer of chitosan to avoid the adsorption. Still, in all immobilization techniques, not more than 24% of the residual activity has been recovered. The least residual activity was observed for the entrapment of the enzyme since organic solvents were used in that method known to inactivate the enzyme. In general, for entrapment—even by other means where stability is maintained—the biggest issue is the transport of oleic acid in aqueous solutions towards the active sites. The chitosan-coated magnetic beads as best candidates were finally chosen for testing rounds of recycling and after 5 cycles, still 70% of initial activity was left. Each reaction of a cycle was performed for 2 h, however, usually, reactions with Ohys with high concentrations of oleic acid take much longer and thus the stability after each cycle might not be the same as shown in that study. Furthermore, it has not been investigated how the activity of immobilized Ohy changes during extended storage for days [37].

Since isolated enzymes are less protected when they are not part of a whole-cell catalyst, their stability and maintenance of activity over a long time plays a crucial role for an efficient process. In a few studies, low stability of Ohys has been observed. Some lose their activity already after a short period of time [25, 37]. In a comparative study on enzyme stability, five different Ohys were analysed. It was found that all of them started to denature already after one day within lysates, leading to an exposure of their hydrophobic sites. As a result, neither substrate nor product was measurable anymore, most likely since they interact with the hydrophobic sites of the denatured protein bulk. Buffer optimization led to certain improvements regarding the protein stability [25]. In another study it was reported that OhyEm loses 60% of its activity already after 7 days at 4 °C. Todea et al. suspected OhyEm to inactivate as a result of the dissociation of subunits [37]. In general, however, not much is published about the stability of Ohys over a longer period of time since most studies have no industrial but rather a medicinal background. Todea et al. have used additives in order to overcome the stability problems. This has been investigated by storing the protein for 7 days at 4 °C and testing its residual activity. However, no experiments have been conducted what effects the additives have on the process stability with several re-usage cycles and at elevated temperatures. Additionally, additives can complicate the process since they might have to be removed before the reaction starts and they increase the price of a process [37]. In general, the main reasons for a loss of protein activity is either the distortion of the tertiary structure, the dissociation of cofactors, chemical inactivation when a reactive chemical is part of the reaction or—as the first step of inactivation for multimeric proteins—the dissociation of subunits [85]. Consequently, multimeric and FAD-bound enzymes are more affected and less advantageous in industrial processes. First of all, a monomeric enzyme can overcome the issues of subunit dissociation and an enzyme working without FAD can’t be subject to cofactor loss. However, currently OhyRe is the only known monomeric Ohy and it loses FAD during purification resulting in a loss of function [48]. Consequently, this particular enzyme still requires optimizations in order to be used in isolated form since the addition of FAD renders it too costly.

### Protein engineering of Ohys

Protein engineering is one of the main methods to overcome the several drawbacks of Ohys. Substrate spectrum and selectivity, cofactor binding, stability and turnover number are attributes, which are desirable to improve. Directed evolution and site-directed mutagenesis are the two main methods for improving proteins.

The crystal structure of OhyEm has been reported with bound FAD, but not with substrate or product [46]. However, an electron density in the proposed substrate binding cavity has been interpreted as a PEG molecule thought to be a substrate mimic. To manipulate the substrate spectrum, structure-guided protein engineering using site-directed mutagenesis has been performed. Hence, amino acids belonging to the pocket of the fatty acid head group were altered. Some of those variants could convert derivatives of oleic acid such as ethyl- and *n-*propyl oleic acid, stearyl alcohol or stearyl amine at higher rates [54]. In another study, the substrate spectrum was altered towards alkenes with a terminal or internal double bond by the addition of a dummy carboxy acid to artificially expand the size of the substrate and by decreasing the size of the substrate binding pocket by mutagenesis. Since the location of PEG was not sufficient, a structure of OhyEm with a docked oleic acid was used [86].

Another attempt to alter the substrate spectrum of Ohys by rational-mutagenesis has been demonstrated by Eser et al*.* In their study, residues of the active side of Ohys with 76% homology originating both from *Lactobacillus acidophilus* have been compared and their functionalities have been estimated by using the crystal structure of OhyEm. One of the enzymes FA-HY2 is unique since it is able to convert substrates up to the length of 22, whereas the other one serves as a rather typical Ohy (FA-HY1) converting a substrate length of 16–18. In conclusion the substrate preference and regioselectivity of FA-HY1 could be changed by swapping critical residues from FA-HY2 [63].

These findings suggest that site-directed mutagenesis has great potential when the crystal structure is fully unravelled and the location of substrates and products is clear or can be cleared by docking experiments. The crystal structure of OhyRe is neither resolved with FAD nor substrate and docking of neither cofactor nor substrate has been successful so far. This can be due to many reasons but a high-quality structure after docking can only be achieved when the underlying biomolecule isn’t subject to large conformational changes upon binding of the docked molecule [87]. That is why directed evolution is sometimes a much more powerful tool specifically for improvements that are difficult to address by structure-based methods, such as melting temperature or affinity towards certain small molecules. Furthermore, results can be achieved at much higher pace. An Ohy from *Paracoccus aminophilus* has been successfully enhanced by using directed evolution. In that context, a coupled assay has been used to screen for optimized variants. 10-HSA was converted into 10-oxostearic acid by an alcohol dehydrogenase, the occurring coenzyme NADH was colorimetrically analysed and the variant with the highest colorimetric output was further analysed. For this type of assay usually performed in lysates the specificity of the alcohol dehydrogenase is crucial [83].

## Conclusions

Ohys belong to the fatty acid hydratases enzyme family, which is unique for its co-factor free modification of free, unsaturated fatty acids. It is suggested, that Ohys primarily evolved to protect microorganisms from toxic effects by incorporation of free, unsaturated fatty acids into the cell membrane. At the beginning of the 1960s also Ohy’s products could be isolated from animal and human faeces, which were assumed to originate from bacteria colonizing the gut. Recently, Ohy evoked industrial interest for the conversion of oleic acid to sustainable 10-HSA, which can replace 12-HSA in oleochemical and cosmetics applications, that is currently generated by hydrogenation of castor oil. Although 12-HSA has a high application spectrum as additive ranging from oils and paints via manufacturing of rubbers to use in food and pharmaceutical industry, the educt castor oil is limited and fluctuates in quality. Furthermore, the hydration process needs high pressure and temperature conditions to obtain economically sound yields. Therefore, industry demands for a more sustainable and quality stable replacement, which can be provided in theory by 10-HSA.

As a consequence, scientists became interested in Ohys, which is documented by an increase in articles characterising Ohys from different microorganisms. While numerous new Ohys have been described recently, there is a limited understanding concerning structure–function relationships in this structurally diverse enzyme family. Specifically, more insights on detailed reaction mechanisms are required. In that context, it is unclear how the highly elongated substrate reaches the active centre and how the hydroxylated products are released after commencement of the reaction. Additionally, a big controversy exists regarding the role of the FAD molecule bound in the structure.

Moreover, with the exception of the monomeric OhyRe recently described, all other Ohys deciphered today are dimers. Hence, OhyRe is amenable for efficient immobilisation, which makes it attractive for industrial applications, which require extended residence times of the biocatalyst in target reactions to reduce costs. However, current literature studies revealed that OhyRe has a significantly different domain architecture to other Ohys, which suggests that this enzyme may follow a different reaction mechanism.

Currently, it is the yet unknown OhyRe reaction mechanism and structure–function relationship of this enigmatic monomeric enzyme as well as the complicated purification process for the target product 10-HSA, which hamper further industrial scaling of the reaction. Therefore, a broader knowledge-base is needed to enable industrial adaptation of biotechnological 10-HSA production to replace thermocatalytic 12-HSA synthesis.

## Supplementary Information

**Additional file 1.** Additional Tables.

## Data Availability

Not applicable.

## Abbreviations

FAD: Binding of flavin adenine dinucleotide
Ohy: Oleate hydratase
PEG: Polyethylene glycol
Rmsd: Root mean square deviation
STY: Space–time yield
