# Supplementary material for: Towards an understanding of oleate hydratases and their application in industrial processes
Source: Microb Cell Fact. 2022 Apr 9;21:58. doi: 10.1186/s12934-022-01777-6 (PMC8994360; doi:10.1186/s12934-022-01777-6)
Supplement: Supplementary file 1 — Additional file 1. Additional Tables. [file 12934_2022_1777_MOESM1_ESM.pdf]

Additional file 1:

Towards an understanding of oleate hydratase and their application in industrial processes

Sophia Prem[1], Carl P. O. Helmer[2], Nicole Dimos[2], Stephanie Himpich[2], Thomas Brück[1], Daniel Garbe[1,*], and Bernhard Loll[2,*]

[1] Werner Siemens-Chair of Synthetic Biotechnology, Dept. of Chemistry, Technical University of Munich (TUM), Lichtenbergstr. 4, 85748 Garching, Germany

[2] Institut für Chemie und Biochemie, Strukturbiochemie, Freie Universität Berlin, Takustr. 6, 14195 Berlin, Germany

* To whom correspondence should be addressed:

Email: daniel.garbe@tum.de

Email: loll@chemie.fu-berlin.de

Table S1 Overview of available structures of Ohys with cofactor or ligand decoration. Assignment to the respective family of oleate hydratases is given according to Schmidt *et al*. [1]. The PEG molecule originates from the crystallization condition. In the structure of OhySa [2], there are three copies in the asymmetric unit, that are described with differently bound substrates or products.

Protein	Organism	HFam Family	Resolution	Cofactor/ Ligand	PDB-ID	Reference
OhyLa	*L. acidophilus*	HFam2	2.22 Å	none	4IA5	[3]
OhyLa	*L. acidophilus*	HFam2	1.80 Å	linoleic acid	4IA6	[3]
OhySa	*S. aureus*	HFam2	1.84 Å	PEG	7KAV	[2]
OhySa	*S. aureus*	HFam2	2.10 Å	FAD / PEG	7KAW	[2]
OhySa (E82A)	*S. aureus*	HFam2	3.51 Å	none	7KAX	[2]
OhySa (E82A)	*S. aureus*	HFam2	1.95 Å	oleate	7KAY	[2]
OhySa (E82A)	*S. aureus*	HFam2	1.85 Å	FAD / oleate / 10-HSA	7KAZ	[2]
OhyRe	*R. erythropolis*	HFam3	2.64 Å	none	5ODO	[4]
OhySt	*Stenotrophomonas* sp. KCTC 12332	HFam11	2.91 Å	none	5Z70	[5]
OhyEm	*E. meningoseptica*	HFam11	2.75 Å	FAD / PEG	4UIR	[3]

Table S2 Pairwise amino acid sequence comparison. Given are identities and in parenthesis the homology. Pairwise alignments were performed with EMBOSS [6].

	OhyLa	OhySa	OhyRe	OhySt
**OhyLa**	-	-	-	-
**OhySa**	61% (76%)	-	-	-
**OhyRe**	33% (48%)	33% (48%)	-	-
**OhySt**	37% (55%)	35% (50%)	27% (42%)	-
**OhyEm**	39% (56%)	41% (56%)	28% (44%)	56% (72%)

Table S3 Results of a DALI search [7] for Ohys with the OhySa [2] as reference structure.

PDB-ID	rmsd [Å]	sequence identity [%]	Z-score	Organism	HFam Family	Cofactor/ Ligand	Reference
7KAZ	0.0	100	66.7	*S. aureus*	HFam2	FAD / oleate	[2]
7KAY	0.6	100	58.2	*S. aureus*	HFam2	oleate	[2]
4IA6	1.1	61	58.0	*L. acidophilus*	HFam2	linoleic acid	[3]
4UIR	2.1	44	48.8	*E. meningoseptica*	HFam11	FAD / PEG	[8]
5ODO	1.7	36	34.4	*R. erythropolis*	HFam3	none	[4]
5Z70	1.9	42	34.1	*Stenotrophomonas* sp. KCTC 12332	HFam11	none	[3]

References

1. Schmid J, Steiner L, Fademrecht S, Pleiss J, Otte KB, Hauer B: Biocatalytic study of novel oleate hydratases. *Journal of Molecular Catalysis B: Enzymatic* 2017.
2. Radka CD, Batte JL, Frank MW, Young BM, Rock CO: Structure and mechanism of *Staphylococcus aureus* oleate hydratase (OhyA). *J Biol Chem* 2021, 296**:**100252.
3. Volkov A, Khoshnevis S, Neumann P, Herrfurth C, Wohlwend D, Ficner R, Feussner I: Crystal structure analysis of a fatty acid double-bond hydratase from *Lactobacillus acidophilus*. *Acta Crystallogr D Biol Crystallogr* 2013, 69**:**648-657.
4. Lorenzen J, Driller R, Waldow A, Qoura F, Loll B, Brück T: *Rhodococcus erythropolis* Oleate Hydratase: a New Member in the Oleate Hydratase Family Tree-Biochemical and Structural Studies. *ChemCatChem* 2018, 10**:**407-414.
5. Park AK, Lee GH, Kim DW, Jang EH, Kwon HT, Chi YM: Crystal structure of oleate hydratase from *Stenotrophomonas* sp. KCTC 12332 reveals conformational plasticity surrounding the FAD binding site. *Biochem Biophys Res Commun* 2018, 499**:**772-776.
6. Rice P, Longden I, Bleasby A: EMBOSS: the European Molecular Biology Open Software Suite. *Trends Genet* 2000, 16**:**276-277.
7. Holm L, Rosenstrom P: Dali server: conservation mapping in 3D. *Nucleic acids research* 2010, 38**:**W545-549.
8. Engleder M, Pavkov-Keller T, Emmerstorfer A, Hromic A, Schrempf S, Steinkellner G, Wriessnegger T, Leitner E, Strohmeier GA, Kaluzna I, et al: Structure-Based Mechanism of Oleate Hydratase from *Elizabethkingia meningoseptica*. *Chembiochem* 2015, 16**:**1730-1734.
